# Evaluation of the real-world implementation of the Family Nurse Partnership in England: an observational cohort study using linked data from health, education and children’s social care.

**DOI:** 10.23889/ijpds.v7i3.1831

**Published:** 2022-08-25

**Authors:** Francesca Cavallaro, Sally Kendall, Jan van der Meulen, Eilis Kennedy, Emma Howarth, Ruth Gilbert, Katie Harron

**Affiliations:** 1 The Health Foundation; 2 University of Kent; 3 London School of Hygiene and Tropical Medicine; 4 The Tavistock and Portman NHS Foundation Trust; 5 University of East London; 6 UCL

## Objectives

The Family Nurse Partnership (FNP) is an early home visiting service supporting young mothers. A randomised controlled trial of FNP in England found no effect on short-term primary outcomes or maltreatment up to age seven, but positive impacts on educational outcomes by age 7. We report preliminary results of an evaluation of FNP using linked administrative data.

## Approach

We constructed a cohort of all mothers aged 13-19 and their firstborn child(ren) giving birth between 2010 and 2017, using linked administrative data from hospital admissions (Hospital Episode Statistics) and education and social care (National Pupil Database). We evaluated differences in a range of policy relevant child and maternal outcomes, comparing mothers who were enrolled in FNP with those who were not using propensity score matching.

## Results

Of 110,960 mothers in our linked cohort, 26,290 (24%) were enrolled in FNP. FNP mothers were younger, more deprived, and more likely to have adversity or social care histories than mothers not enrolled. Preliminary results suggest that FNP was not associated with fewer unplanned hospital admissions for injury / maltreatment by age two, improved child development at age 4, persistent school absence or children looked after in out-of-home care by age 7, or improved maternal outcomes. Some adverse outcomes appeared to be increased in the FNP group. We will present findings amongst subgroups of younger maternal age (13-15 years), increased deprivation according to quintile of Index of Multiple Deprivation, and adversity and social care history. We also present sensitivity analyses that aim to minimise confounding.

## Conclusion

Our study supports findings from previous trials of FNP showing little benefit for measured child and maternal outcomes. Interpretation of results needs careful consideration of the impact of residual confounding due to unmeasured or undisclosed factors (e.g. family violence) linked to targeting of FNP to higher risk mothers, and surveillance bias.

